# miR-301a-3p induced by endoplasmic reticulum stress mediates the occurrence and transmission of trastuzumab resistance in HER2-positive gastric cancer

**DOI:** 10.1038/s41419-021-03991-3

**Published:** 2021-07-13

**Authors:** Jing Guo, Xuxian Zhong, Qinglin Tan, Shengnan Yang, Jiaqi Liao, Jinke Zhuge, Ziyang Hong, Qiong Deng, Qiang Zuo

**Affiliations:** 1 Department of Oncology, Nanfang Hospital, Southern Medical University, Guangzhou, Guangdong Province China 510515; 2 Department of Internal Medicine, Guangdong Provincial People’s Hospital, Guangdong Academy of Medical Sciences, Guangzhou, Guangdong Province China 510080; 3 Department of Oncology, Dongguan People’s Hospital, Southern Medical University, Dongguan, Guangdong Province China 523059

**Keywords:** Cancer therapeutic resistance, Gastric cancer

## Abstract

Trastuzumab resistance negatively influences the clinical efficacy of the therapy for human epidermal growth factor receptor 2 (HER2) positive gastric cancer (GC), and the underlying mechanisms remain elusive. Exploring the mechanisms and finding effective approaches to address trastuzumab resistance are of great necessity. Here, we confirmed that endoplasmic reticulum (ER) stress-induced trastuzumab resistance by up-regulating miR-301a-3p in HER2-positive GC cells. Moreover, we elucidated that miR-301a-3p mediated trastuzumab resistance by down-regulating the expression of leucine-rich repeats and immunoglobulin-like domains containing protein 1 (LRIG1) and subsequently activating the expression of insulin-like growth factor 1 receptor (IGF-1R) and fibroblast growth factor receptor 1 (FGFR1) under ER stress. We also found that intercellular transfer of miR-301a-3p by exosomes disseminated trastuzumab resistance. The present study demonstrated that exosomal miR-301a-3p could serve as a non-invasive biomarker for trastuzumab resistance, which was maybe a novel potential therapeutic target to overcome trastuzumab resistance and improve the curative effect of trastuzumab in HER2-positive GC patients.

## Introduction

Gastric cancer (GC) is the 5th most commonly diagnosed malignancy and the 3rd leading cause of cancer mortality around the world [1]. Human epidermal growth factor receptor 2 (HER2) amplification or overexpression affects ~6.1–23.0% of GC patients [2]. In comparison with using chemotherapy alone, ToGA trial showed that adding trastuzumab (Herceptin, Roche), an effective anti-HER2 humanized monoclonal antibody, in traditional chemotherapy could noticeably increase progression-free survival and overall survival in HER2-positive GC [3]. In patients with HER2-positive GC, therapies combining with trastuzumab have become a standard first-line treatment. However, some patients could not receive satisfactory effects after these therapies, and cancer progressed rapidly in three to four months with a poor prognosis [3, 4]. Currently, the underlying molecular mechanism of trastuzumab resistance in HER2-positive GC is still unclear, and the availability of surrogate markers to predict resistance remains an unmet need.

Endoplasmic reticulum (ER) stress is induced by the accumulating misfolded or unfolded proteins, which increase in response to micro-environmental stimuli [5]. When ER stress occurs, self-protective signalling transduction pathways, termed as unfolded protein response (UPR), are developed to restore homeostasis or activate cell death. The UPR is initiated when the ER chaperone molecule glucose-regulated protein 78 (GRP78) induces the activation of downstream proteins. ER stress was identified in a variety of tumours, and had a pivotal part in tumour initiation, development and drug resistance. Moreover, chemotherapy resistance in solid tumours was previously found to be related to transmissible ER stress [6].

Exosomes, a kind of small extracellular vesicles, contain lipids, proteins, and genetic substances. They can be detected in body fluids and range in size from 30 to 150 nm [7]. Exosomes can be secreted by various types of cells, including cancer cells. Exosomes function in intercellular communication via the transmission of cargoes in the tumour microenvironment [7, 8]. Recent studies had indicated that exosome release was increased during ER stress [9–12] and UPR induction enhanced exosome cargo [13]. An increasing amount of evidence implied that exosomes had a pivotal part in promoting tumour development via mediating angiogenesis and metastasis [14, 15]. It has been indicated that microRNAs (miRNAs) are commonly enriched in exosomes [16, 17] and exosomal miRNAs could transmit between cells and exerted regulatory effects on drug resistance in multiple cancers [18–20]. Nevertheless, it still remains unclear if there are specific exosomal miRNAs regulating trastuzumab resistance, which can be applied as a non-invasive biomarker for HER2-positive GC.

In this study, we found that miR-301a-3p induced by ER stress-mediated trastuzumab resistance through activating the expression of insulin-like growth factor 1 receptor (IGF-1R) and fibroblast growth factor receptor 1 (FGFR1) via directly targeting immunoglobulin-like domains containing protein 1 (LRIG1). Moreover, intercellular transfer of miR-301a-3p by exosomes disseminated trastuzumab resistance. These results could provide a reliable biomarker for monitoring and a new therapeutic target for reducing trastuzumab resistance in treating patients with HER2-positive GC.

## Results

### Trastuzumab resistance was related to ER stress in HER2-positive GC

**Fig. 1:**
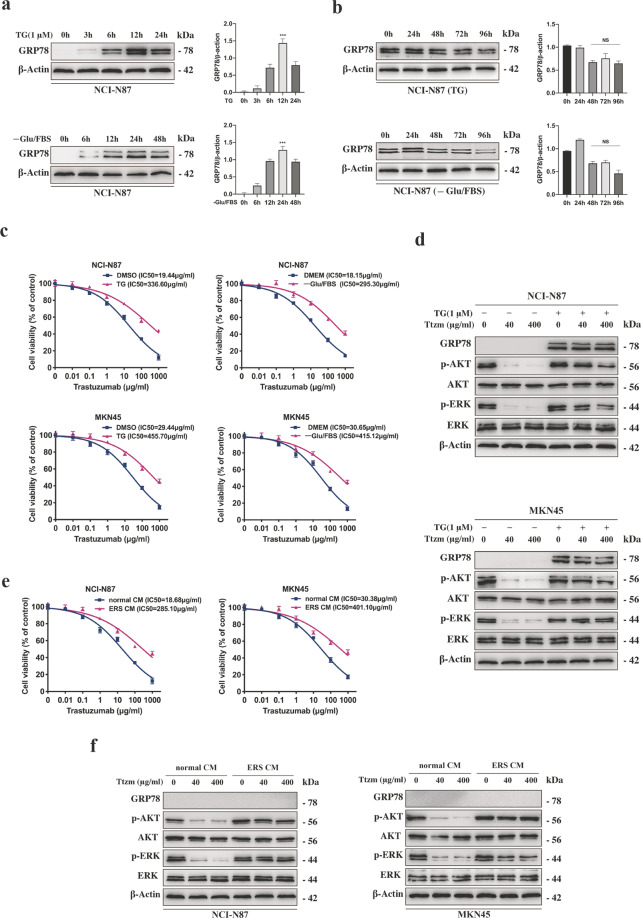
Activation of ER stress in HER2-positive GC cells was related to trastuzumab resistance. **a** Representative western blot and quantification for GRP78 in NCI-N87 cells after treated with 1 μM TG for 0, 3, 6, 12 and 24 h and -Glu/FBS for 0, 6, 12, 24 and 48 h, respectively. **b** Representative western blot and quantification for GRP78 in NCI-N87 cells after treated with 1 μM TG for 12 h or -Glu/FBS for 24 h following culture in normal CM for 0, 24, 48, 72, and 96 h, respectively. **c** CCK8 assay after NCI-N87 and MKN45 cells were treated by 1 μM TG for 12 h or -Glu/FBS for 24 h before trastuzumab treatment for 72 h (*n* = 3). **d** Western blot analysis for protein expression in NCI-N87 and MKN45 cells after treatment with or without 1 μM TG for 12 h followed by 40 or 400 μg/ml trastuzumab treatment for 72 h. **e** CCK8 assay after NCI-N87 and MKN45 cells were treated by specific medium for 24 h before trastuzumab treatment for 72 h (*n* = 3). **f** Western blot analysis for protein expression in NCI-N87 and MKN45 cells after treatment with specific medium for 24 h after 40 or 400 μg/ml trastuzumab treatment for 72 h. Statistical analysis was performed by Student’s *t*-test, error bars indicate SD (***p < 0.001, NS no significance).

To study whether trastuzumab resistance has resulted from ER stress in HER2-positive GC cells, NCI-N87 was incubated with the ER stress inducer thapsigargin (TG) or -Glu/FBS. GRP78 expression was analysed by conducting western blot at each time point (Fig. 1a). Even if ER stressed NCI-N87 cells were incubated with a normal culture medium, the UPR effect was still long-lasting (Fig. 1b). ER stress didn’t affect the HER2 expression level of HER2 positive and negative GC cells (Supplementary Fig. S1a). As shown by the curve of concentration effects (Fig. 1c), the IC50 value of trastuzumab for ER stressed or unstressed cells indicated that ER stressed cells were ten times more resistant to trastuzumab compared with unstressed cells. Besides, the number of apoptotic cells in ER stressed cells was reduced as compared to unstressed cells (Supplementary Fig. S1b). We validated that phosphorylated extracellular signal-regulated kinase (p-ERK) and phosphorylated protein kinase B (p-AKT) levels were higher in ER stressed cells compared with unstressed cells after treatment with low or high doses of trastuzumab (Fig. 1d, Supplementary Fig. S1c). We also analysed the intracellular calcium level and the time-dependent induction of HER2, p-ERK and p-AKT expression during ER stress (Supplementary Fig. S1d–e). The intracellular calcium level was increased in GC cells incubated with TG, but no change with -Glu/FBS. The results of these two treatments making GC cells resistant to trastuzumab samely indicated that the intracellular calcium level had no effect on trastuzumab resistance induced by ER stress. Next, we prepared ER stress (ERS) conditioned medium (CM) using GC cells cultured with TG. It was found that unstressed NCI-N87 and MKN-45 cells became resistant to trastuzumab after incubation with ERS CM, whereas there was no ER stress transmission (Fig. 1e–f). These data suggested that the transmission of trastuzumab resistance was not due to transmissible ER stress.

### ER stress-mediated trastuzumab resistance by inducing the overexpression of miR-301a-3p

**Fig. 2:**
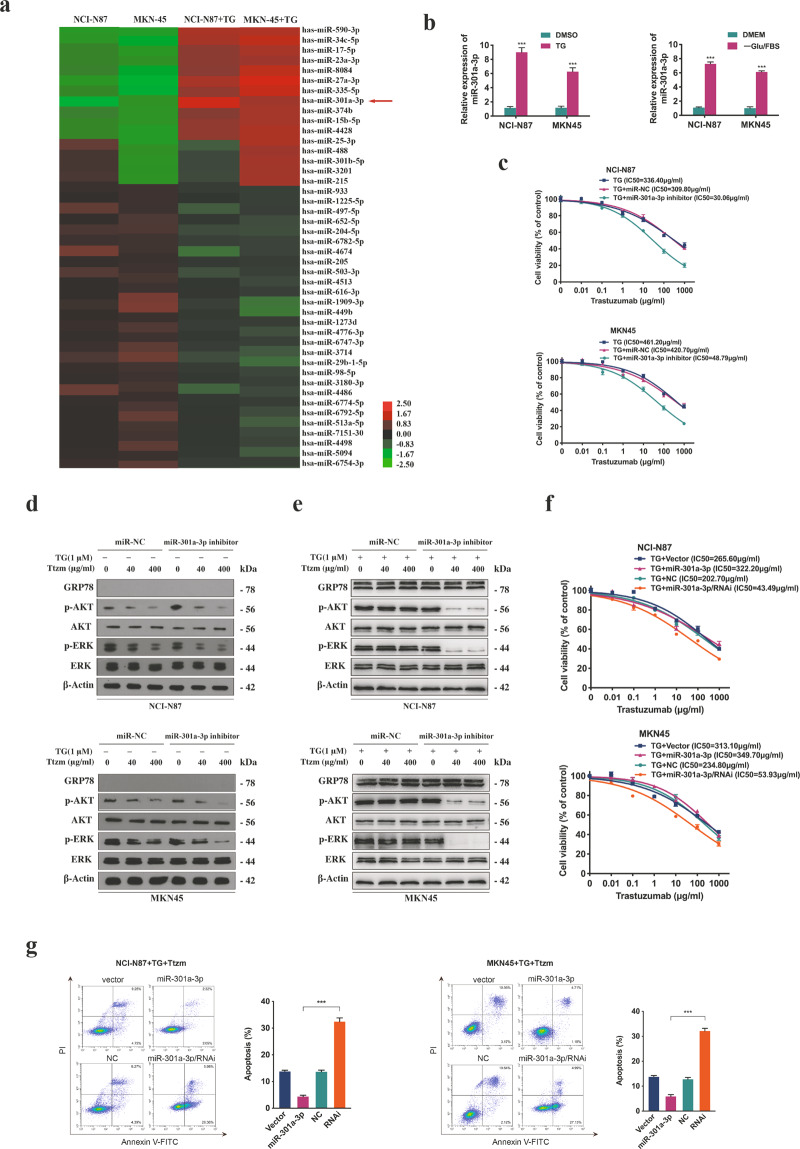
Identification of miR-301a-3p and the effect on trastuzumab resistance. **a** Microarray assay of NCI-N87 and MKN45 cells which were treated with or without 1 μM TG for 12 h. Hierarchical clustering of the expression levels of miRNAs in cells. Red and green represent high or low relative expression, respectively. Increased and decreased miRNAs levels were determined by *P* < 0.05 and >2.5 folds difference in expression compared with the blank control. The arrow represents miR-301a-3p. **b** The relative expression level of miR-301a-3p level in NCI-N87 and MKN45 cells treated with 1 μM TG for 12 h or -Glu/FBS for 24 h. **c** CCK8 assay of NCI-N87 and MKN45 cells after transfection with miR-301a-3p inhibitor or control agents before trastuzumab treatment at indicated concentrations for 72 h (*n* = 3). **d** Protein level in NCI-N87 and MKN45 cells after transfection with miR-301a-3p inhibitors or control followed by 40 or 400 μg/ml trastuzumab treatment for 72 h. **e** The certain proteins in NCI-N87 and MKN45 cells after transfection with miR-301a-3p inhibitors or control detected by Western blot. The cells were treated with 1 μM TG for 12 h followed by 40 or 400 μg/ml trastuzumab treatment for 72 h. **f** CCK8 assay of miR-301a-3p stably transfected and control cells. The NCI-N87 and MKN45 cells were treated with 1 μM TG for 12 h followed by trastuzumab treatment for 72 h (*n* = 3). **g** Flow cytometry was used to measure the apoptosis of miR-301a-3p stably transfected and control cells after treated with 1 μM TG for 12 h followed by 400 μg/ml trastuzumab treatment for 72 h (*n* = 3). Statistical analysis was performed by Student’s *t*-test, error bars indicate SD (***p < 0.001).

To find out the potential resistance mechanisms, we analysed the miRNA expression profile of ER stressed and unstressed GC cells by miRNA microarray analysis (Fig. 2a). It was suggested that 11 miRNAs were remarkably upregulated (>2.5 folds) in ER stressed cells compared with unstressed cells, and qRT-PCR was used to validate the differentially expressed miRNAs (Supplementary Fig. S2a). To identify the miRNAs related to trastuzumab resistance, we conducted a loss-of-function analysis of 11 selected miRNAs in ER stressed cells by miRNA inhibitors. Notably, interference with miR-301a-3p reversed trastuzumab resistance in comparison with the remaining ten miRNAs (Supplementary Fig. S2b). The miR-301a-3p level was remarkably elevated in ER stressed cells (Fig. 2b, Supplementary Fig. S2c), but no significant change of p-Argonaute 2 (p-Ago2) level was found (Supplementary Fig. S2d). Moreover, the miR-301a-3p expression at a high level was detected even in ER stressed cells incubated with normal CM (Supplementary Fig. S2e). Silencing miR-301a-3p in ER stressed cells contributed to a markedly reduced IC50 value and potent suppression on ERK and AKT signal pathways after treatment with trastuzumab (Fig. 2c–e, Supplementary Fig. S2f)). To verify the effects of miR-301a-3p on trastuzumab resistance, we established stably transfected GC cells by over-expressing and suppressing (RNAi) the expression of miR-301a-3p, while HER2 level showed no significant change (Supplementary Fig. S2g–h). Moreover, inhibiting the miR-301a-3p expression of ER stressed cells help reduce IC50 value and increase cell apoptosis rate after trastuzumab treatment (Fig. 2f–g, Supplementary Fig. S2i). These results demonstrated that upregulated miR-301a-3p induced by ER stress was responsible for trastuzumab resistance.

### miR-301a-3p induced by ER stress-mediated trastuzumab resistance by downregulating the expression of LRIG1

**Fig. 3:**
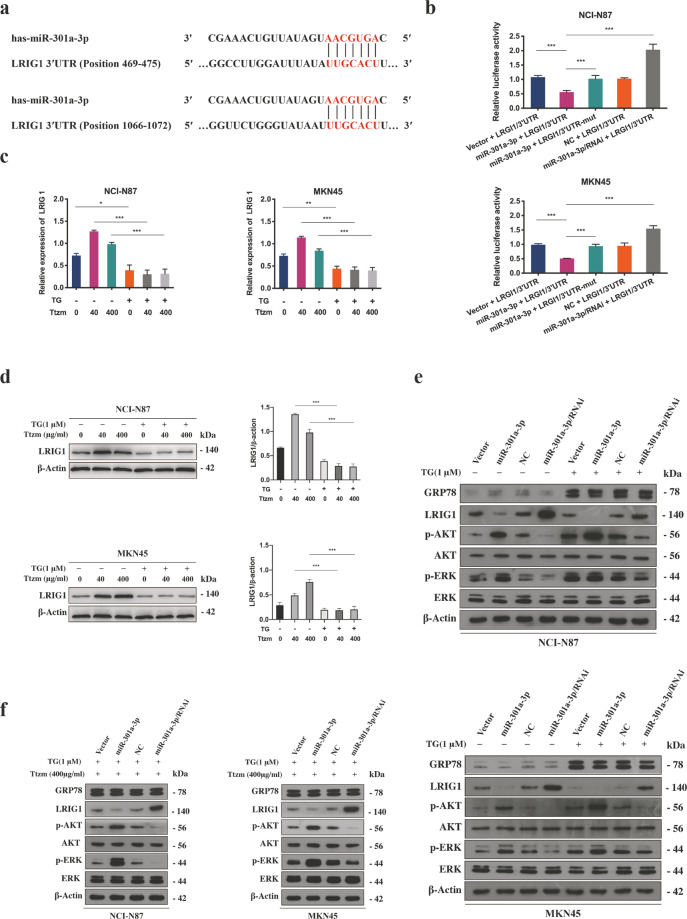
miR-301a-3p mediated trastuzumab resistance by downregulating LRIG1 expression via directly targeting its 3′-UTR in ER stressed GC cells. **a** The bioinformatics analyses of TargrtScan7.1 and miRDB databases indicated that the 3′-UTR sequence of LRIG1 mRNA contained two binding sites of miR-301a-3p at nucleotide positions of 469-475 and 1066-1072. **b** The miR-301a-3p mimics, or NC and the luciferase reporter plasmids containing wide type or mutant 3′-UTR region of LRIG1 were co-transfected into NCI-N87 and MKN45 cells. Relative luciferase activity was then detected. (*n* = 3). **c** qRT-PCR analysis for the LRIG1 mRNA in NCI-N87 and MKN45 cells treated with or without 1 μM TG for 12 h followed by 40 or 400 μg/ml trastuzumab treatment for 72 h (*n* = 3). **d** Representative western blot and quantification for LRIG1 in NCI-N87 and MKN45 cells treated with or without 1 μM TG for 12 h following by 40 or 400 μg/ml trastuzumab treatment for 72 h. **e** The indicated proteins of NCI-N87 and MKN45 cells, which were stably transfected with miR-301a-3p or control and treated with or without 1 μM TG for 12 h were detected by western blot. **f** The indicated proteins of NCI-N87 and MKN45 cells, which were stably transfected with miR-301a-3p or control and treated with 1 μM TG for 12 h followed by 400 μg/ml trastuzumab treatment for 72 h were detected by western blot. Statistical analysis was performed by Student’s *t*-test, error bars indicate SD (*p < 0.05, **p < 0.01, ***p < 0.001).

To find out the molecular mechanism involved in miR-301a-3p mediating trastuzumab resistance, bioinformatics analyses were applied to discover potential targets of miR-301a-3p by using TargrtScan7.1 and miRDB databases. The results indicated that one of the targets was LRIG1 and that the 3′-untranslated region (3′-UTR) of LRIG1 mRNA contained two binding sites at nucleotide positions 469-475 and 1066-1072 (Fig. 3a).

To verify the direct binding relationship between LRIG1 and miR-301a-3p, a luciferase reporter assay was conducted with luciferase reporter plasmid containing wild-type and mutant 3′-UTR of LRIG1 including miR-301a-3p binding site. As demonstrated in Fig. 3b, the luciferase activity in wild-type Luc-LRIG1-3′-UTR-transfected cells was remarkably decreased by miR-301a-3p in comparison with the LRIG1 3′-UTR mutant cells. We also detected the LRIG1 expression in GC cells after culture with TG or -Glu/FBS at each time point and its expression showed a downward trend with the prolongation of the treatment time (Supplementary Fig. S1c, Fig. S3c).

Furthermore, we observed obviously decreased expression of LRIG1 in ER stressed GC cells compared with unstressed GC cells after treatment with low or high doses of trastuzumab (Fig. 3c–d). Silencing miR-301a-3p in ER stressed GC cells led to significantly increased expression of LRIG1 and downregulated p-AKT and p-ERK compared with the cells with overexpressed miR-301a-3p (Fig. 3e). In addition, miR-301a-3p knockdown contributed to the overexpression of LRIG1 and suppression of ERK and AKT signal after trastuzumab treatment, suggesting that inhibiting miR-301a-3p restored the trastuzumab response in ER stressed GC cells (Fig. 3f). These results implied that miR-301a-3p directly targeted LRIG1 and was inversely correlated with LRIG1 levels in ER stressed GC cells.

### LRIG1 regulated by miR-301a-3p mediated trastuzumab resistance by activating the expression of IGF-1R and FGFR1

**Fig. 4:**
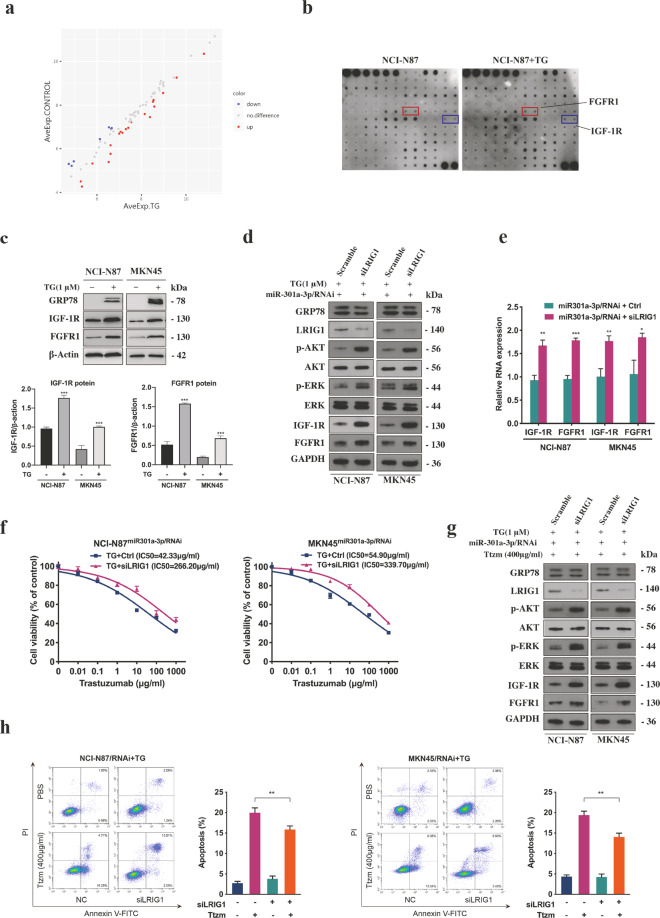
LRIG1 regulated by miR-301a-3p mediated trastuzumab resistance through activating the expression of IGF-1R and FGFR1. **a** Differential expression proteins. The basic statistic used for significance analysis is fold change. Results include (log2) the fold changes of every protein and its contrast individually. Scatter plot: red presents upregulation, blue presents downregulation, and grey presents no difference. **b** Whole-cell extracts from NCI-N87 cells after treated with or without 1 μM TG for 12 h were incubated in the arrays of RTK antibody. Phosphorylated proteins were measured by incubating with anti-phosphotyrosine horseradish peroxidase. **c** Representative western blot and quantification for IGF-1R and FGFR1 in NCI-N87 and MKN45 cells after treated with or without 1 μM TG for 12 h. **d** Western blot analysis of indicated proteins in miR-301a-3p knockdown NCI-N87 and MKN45 cells treated with 1 μM TG for 12 h followed by transfection with LRIG1 siRNAs (siLRIG1) or NC. **e** qRT-PCR analysis of IGF-1R and FGFR1 mRNA in miR-301a-3p knockdown NCI-N87 and MKN45 cells after transfection with siLRIG1 or control (*n* = 3). **f** CCK8 assay of ER stressed miR-301a-3p knockdown NCI-N87 and MKN45 cells transfected with siLRIG1 or control before or control and treated with TG and followed by trastuzumab treatment (*n* = 3). **g** Western blot analysis of certain proteins in ER stressed miR-301a-3p knockdown NCI-N87 and MKN45 cells transfected with siLRIG1 or control followed by 400 μg/ml trastuzumab treatment for 72 h. **h** Flow cytometry was used to measure the apoptosis of ER stressed miR-301a-3p knockdown NCI-N87 and MKN45 cells transfected with siLRIG1 or control after exposure to 400 μg/ml trastuzumab for 72 h (*n* = 3). Statistical analysis was performed by Student’s *t*-test, error bars indicate SD (*p < 0.05, **p < 0.01, ***p < 0.001).

LRIG1 is an important negative regulator of receptor protein tyrosine kinase (RTK). It has been reported that the overexpression of RTKs was closely related to trastuzumab resistance [21]. To further study how LRIG1 mediated trastuzumab resistance, we tested RTK phosphorylation levels in ER stressed and unstressed GC cells using phospho-RTK array experiment. As shown in Fig. 4a, there were 27 differentially expressed proteins (DEPs) and their fold changes were <0.83 or more than 1.2 (absolute logFC > 0.263). DEPs were then categorized according to molecular function, biological process and cellular component using gene ontology (GO) annotation. In KEGG analysis, most DEPs were related to PI3K-AKT and mitogen-activated protein kinase (MAPK) signalling pathways (Supplementary Fig. S4). The results revealed that there were increased expression levels of FGFR1 and IGF-1R in ER stressed GC cells compared with unstressed GC cells (Fig. 4b–c). The knockdown of LRIG1 in ER stressed GC cells with stably silenced miR-301a-3p significantly upregulated the levels of IGF-1R and FGFR1 (Fig. 4d–e). In addition, the inhibition of LRIG1 expression recapitulated the trastuzumab resistance phenotype, decreased cell apoptosis rate and activated downstream AKT and ERK signal after treatment with trastuzumab in ER stressed GC cells with stably silenced miR-301a-3p (Fig. 4f–h, Supplementary Fig. S4e).

### ER stress transmitted trastuzumab resistance by releasing exosomes

**Fig. 5:**
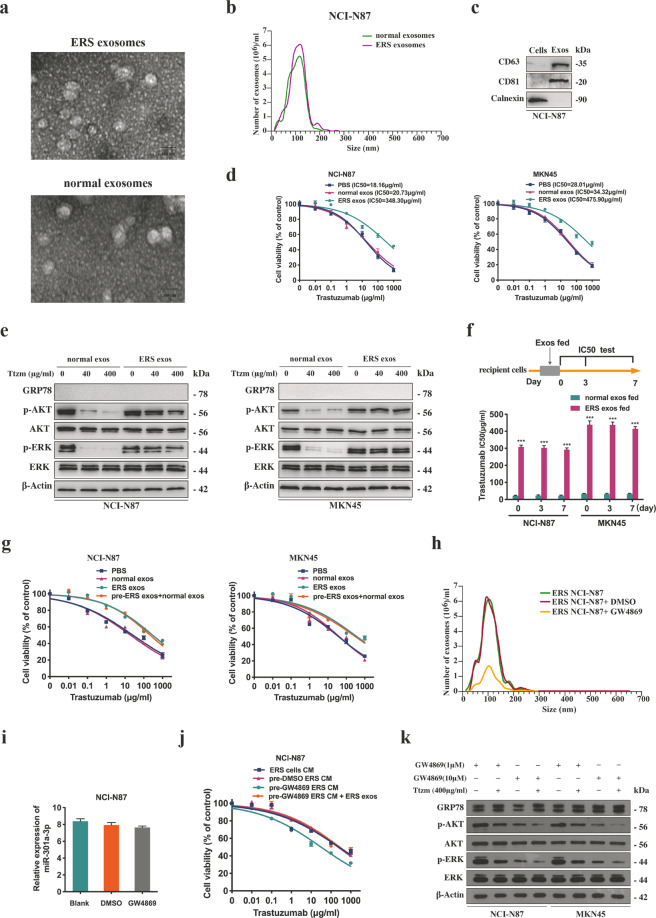
ER stress transmitted trastuzumab resistance by releasing exosomes. **a** Electron microscopy images of exosomes secreted by NCI-N87 cells following treatment with or without 1 μM TG for 12 h. Scale bar: 100 nm. **b** Nanosight particle tracking was used to analyze the sizes and counts of the exosomes secreted by NCI-N87 cells after treated with or without 1 μM TG for 12 h. **c** CD36 and CD81, the markers for exosomes, were detected in exosomes secreted by NCI-N87 cells after treated with 1 μM TG for 12 h by western blot. **d** CCK8 assay of NCI-N87 and MKN45 cells treated by certain exosomes (PBS as control) for 48 h before trastuzumab treatment for 72 h (*n* = 3). **e** Western blot analysis of certain proteins in NCI-N87 and MKN45 cells treated by indicated exosomes for 48 h before trastuzumab treatment for 72 h. **f** NCI-N87 and MKN45 cells were cultured with indicated exosomes for 48 h. IC50 value of trastuzumab on the 0, 3rd, 7th day was detected by CCK8 assay, respectively (*n* = 3). **g** After treatment of ER stressed exosomes for 48 h, MKN45 and NCI-N87 cells were then incubated with normal exosomes, and subjected to CCK8 assay upon trastuzumab treatment for 72 h (*n* = 3). **h** Nanosight particle tracking was used to analyze the size and counts of the exosomes secreted by the NCI-N87 cells treated by 1 μM TG for 12 h followed by 10 μM GW4869 treatments for 24 h. **i** qRT-PCR analysis of miR-301a-3p in NCI-N87 cells after treatment with 1 μM TG for 12 h followed by 10 μM GW4869 treatments for 24 h (*n* = 3). **j** CCK8 assay of the NCI-N87 cells treated with different sources of CM for 24 h before trastuzumab treatment for 72 h (*n* = 3). **k** Western blot analysis of certain proteins in MKN45 and NCI-N87 cells. Cells were treated with different sources of CM with or without 1 or 10 μM GW4869 treatment for 24 h, and then treated by 400 μg/ml trastuzumab for 72 h. Statistical analysis was performed by Student′s *t*-test and one-way ANOVA, error bars indicate SD (***p < 0.001).

Exosomes were extracted from the medium of ER stressed and unstressed GC cells. Purified exosomes appeared as small round vesicles and the diameters were 80–120 nm (Fig. 5a–b). CD63 and CD81 were the markers expressed by exosomes (Fig. 5c). Trastuzumab was treated with the GC cells after co-culture with exosomes from ER stressed or unstressed GC cells. We found that incubation with exosomes from ER stressed cells increased the proliferation, decreased and activated survival-associated signal of unstressed cells in comparison with the control group treated by trastuzumab (Fig. 5d–e). Moreover, the transmitted resistance in the recipient cells could maintain for more than 7 days after ER stressed exosomes were removed (Fig. 5f) and normal exosomes could not reverse this effect (Fig. 5g). It was revealed that the resistance of the recipient cells transmitted by ER stressed exosomes could last for a long time.

Next, we reduced exosome secretion by using GW4869, an agent suppressing exosomes secretion via inhibiting miRNA and nSMase2 [22]. The number of exosomes was markedly decreased in GW4869-treated ER stressed GC cells in comparison with the blank control cells (Fig. 5h), but there was no influence on the intracellular miR-301a-3p levels (Fig. 5i). Furthermore, CM from ER stressed GC cells treated with GW4869 could not transmit trastuzumab resistance to the recipient cells (Fig. 5j–k). The results proved the critical function of exosomes in the transfer of trastuzumab resistance.

### Exosomes released by ER stress transmitted trastuzumab resistance through transporting miR-301a-3p in vitro

**Fig. 6:**
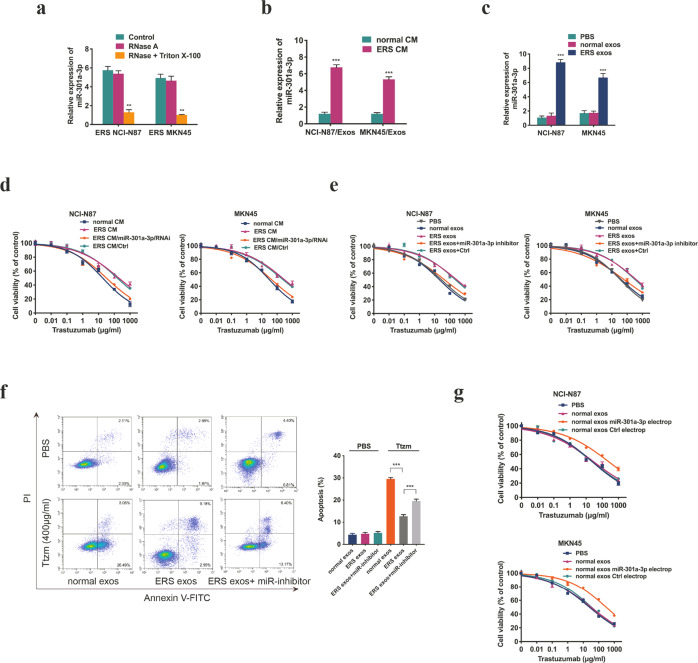
Exosomes released by ER stress transmitted trastuzumab resistance through transporting miR-301a-3p in vitro. **a** qRT-PCR analysis of miR-301a-3p in the CM of NCI-N87 and MKN45 cells treated by 1 μM TG for 12 h following by treatment of RNase (2 mg/ml) only or a combination of RNase and Triton X-100 (0.1%) for 20 minutes (*n* = 3). **b** qRT-PCR analysis of miR-301a-3p in exosomes extracted from the CM of NCI-N87 and MKN45 cells treated with or without 1 μM TG for 12 h (*n* = 3). **c** qRT-PCR analysis of miR-301a-3p in NCI-N87 and MKN45 cells after the cells were incubated with certain exosomes (PBS as control) for 48 h (*n* = 3). **d** CCK8 assay of NCI-N87 and MKN45 cells treated by different sources of CM for 24 h before trastuzumab treatment for 72 h (*n* = 3). **e** CCK8 assay of NCI-N87 and MKN45 cells first incubated with indicated exosomes and transfected with miR-301a-3p inhibitors or NC before trastuzumab treatment at indicated concentrations for 72 h (*n* = 3). **f** Flow cytometry was used to measure the apoptosis of NCI-N87 and MKN45 cells incubated with indicated exosomes and transfected with miR-301a-3p inhibitors followed by 400 μg/ml trastuzumab treatment for 72 h (*n* = 3). **g** Exosomes isolated from NCI-N87 and MKN45 cells were electroporated with miR-301a-3p mimics or control. NCI-N87 and MKN45 cells were cultured with certain exosomes for 48 h and then subjected to CCK8 assay upon trastuzumab treatment at indicated concentrations for 72 h (*n* = 3). Statistical analysis was performed by Student’s *t*-test and one-way ANOVA, error bars indicate SD (**p < 0.01, ***p < 0.001).

Next, we studied whether miR-301a-3p was specifically packaged into exosomes. The miR-301a-3p level in the medium of ER stressed GC cells was slightly changed after treatment with RNase but remarkably reduced after synchronous treatment with Triton X-100 and RNase (Fig. 6a), suggesting that extracellular miR-301a-3p was mainly packed in membranes. The miR-301a-3p level was markedly increased in exosomes secreted by ER stressed GC cells compared with unstressed GC cells (Fig. 6b). Besides, the intracellular miR-301a-3p level was elevated after treatment with the exosomes from ER stressed GC cells (Fig. 6c). CCK8 assay showed that unstressed cells became insensitive to trastuzumab after incubation with ERS CM, whereas ERS CM from miR-301a-3p knockdown GC cells reversed this effect (Fig. 6d). In addition, we found that unstressed cells directly incubated with exosomes from ER stressed GC cells showed decreased sensitivity to trastuzumab and decreased cell apoptosis rate, which was reversed by miR-301a-3p inhibitors in recipient cells (Fig. 6e–f). As shown in Fig. 6g, unstressed GC cells incubated with the exosomes electroporated with miR-301a-3p showed an unsatisfactory response to trastuzumab. These findings suggested that the exosomes from ER stressed GC cells might transmit trastuzumab resistance to unstressed recipient cells via the intercellular transportation of miR-301a-3p.

### Exosomes released by ER stress transmitted trastuzumab resistance through transporting miR-301a-3p in vivo

**Fig. 7:**
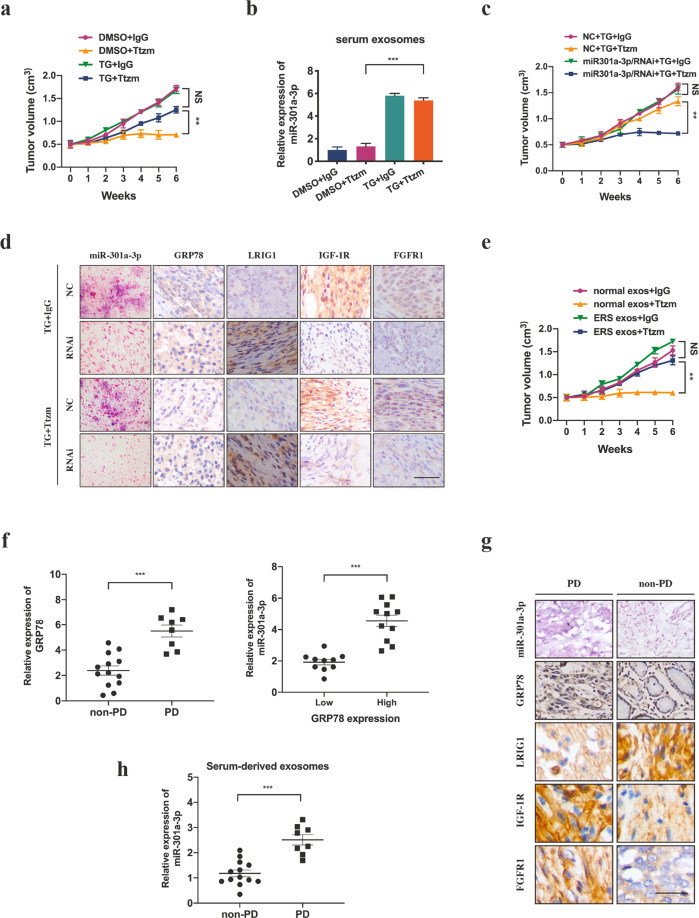
miR-301a-3p induced by ER stress mediates trastuzumab resistance in vivo and in HER2-positive GC patients. **a** Subcutaneous xenograft assay of NCI-N87 cells in mice with subcutaneous injection of TG or DMSO upon trastuzumab or control IgG treatment for indicated days. The volumes of the tumours were shown (*n* = 5 in each group). **b** qRT-PCR analysis of serum exosomal miR-301a-3p level from mice by different treatments *n* = 3. **c** Subcutaneous xenograft assay of NCI-N87 cells in the mice with subcutaneous injection of certain exosomes and trastuzumab or control IgG treatment for indicated days. The volumes of the tumours were shown (*n* = 5 in each group). **d** Representative ISH images of miR-301a-3p and IHC images of indicated proteins in tumour sections of different groups (*n* = 5 in each group). Scale bar: 50 μm. **e** Subcutaneous xenograft assay of NCI-N87 cells in the mice with subcutaneous injection of certain exosomes and trastuzumab or control IgG treatment for indicated days. The volumes of the tumours were shown (*n* = 5 in each group). **f** GRP78 mRNA level of HER2-positive GC patients suffering from PD or non-PD after trastuzumab therapy and miR-301a-3p expression level of HER2-positive GC patients with high or low GRP78 expression. **g** Representative ISH images of miR-301a-3p and IHC images of indicated proteins in tumour sections from HER2-positive GC patients suffering from PD or non-PD after trastuzumab therapy. Scale bar: 50 μm. **h** miR-301a-3p expression level of serum-derived exsomes in HER2-positive GC patients suffering from PD or non-PD after trastuzumab therapy. Statistical analysis was performed by Student’s *t*-test and one-way ANOVA, error bars indicate SD (**p < 0.01, ***p < 0.001).

In the in vivo experiment, NCI-N87 cells were subcutaneously injected into nude mice and either TG or DMSO was interperitoneally injected. It was found that the tumour volume in mice co-treated with TG and trastuzumab was larger than that in the mice co-treated with DMSO and trastuzumab (Fig. 7a, Supplementary Fig. S5a). In addition, the serum exosomal miR-301a-3p level was significantly higher in mice treated with TG than that treated with DMSO (Fig. 7b). Next, the mice were subcutaneously injected with miR-301a-3p knockdown or control cells and treated with TG and trastuzumab during the experiment. Results showed in Fig. 7c and Supplementary Fig. S5a, tumours in the miR-301a-3p knockdown group grew slower than those in the control group. We next performed immunohistochemistry (IHC) and in situ hybridization (ISH) on xenograft tissues, high expression LRIG1, and low expression IGF-1R and FGFR1 were observed in the miR-301a-3p knockdown group (Fig. 7d). Exosomes from ER stressed and unstressed GC cells were intratumorally injected into GC xenografts. In Fig. 7e and Supplementary Fig. S5b, exosomes excreted by ER stressed cells remarkably increased the resistance of GC xenografts to trastuzumab.

### miR-301a-3p induced by ER stress mediates trastuzumab resistance in HER2-positive GC patients

To evaluate the role of ER stress in trastuzumab response in clinic, we assessed a total of 21 tissue samples derived from HER2-positive GC patients treated with trastuzumab, including eight cases evaluated as progressive disease (PD) and 13 as partial response (PR) or stable disease (SD) according to response evaluation criteria in solid tumours version 1.1 (RECIST 1.1). The results indicated the high expression of GRP78 in patients who suffered from PD and the expression of miR-301a-3p was significantly correlated with GRP78 level (Fig. 7f–g). Consistently, low expression of LRIG1, and high expression of IGF-1R and FGFR1 were observed in PD patients (Fig. 7g). Further, we assessed the value of miR-301a-3p in exosomes isolated from the serum of these patients. Exosomal miR-301a-3p was enriched in the serum of patients who suffered from PD after trastuzumab therapy (Fig. 7h). These results indicate that circulating exosomal miR-301a-3p may correlate with the rastuzumab resistance in HER2-positive GC patients.

**Fig. 8:**
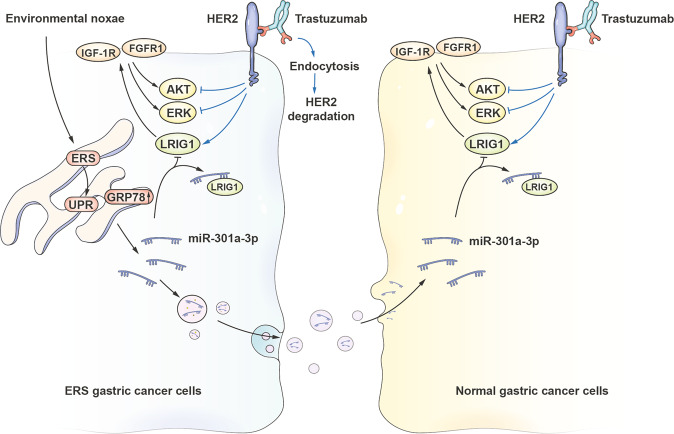
A schematic diagram of miR-301a-3p based signalling circuit of trastuzumab resistance in HER2-positive GC. miR-301a-3p promoted trastuzumab resistance in ER stressed GC cells by downregulating the expression of LRIG1, resulting in an increase in the expression of IGF-1R and FGFR1, and the activation of ERK and AKT signalling pathway. Moreover, miR-301a-3p could be carried by exosomes and secreted by ER stressed GC cells, transmitting trastuzumab resistance to unstressed recipient cells.

In conclusion, ER stressed GC cells produced the exosomes containing miR-301a-3p which could be absorbed by unstressed GC cells, and subsequently enhance the trastuzumab resistance phenotype. This enhanced effect was possibly related to the inhibition of LRIG1 and subsequent facilitation the expression of IGF-1R and FGFR1. To sum up, a pattern diagram of the mechanism function of exosomal miR-301a-3p in HER2-positive GC cells was established (Fig. 8).

## Discussion

Trastuzumab resistance is an obstacle to effective therapy for GC patients. Investigating the mechanisms of the drug resistance might be conducive to develop effective therapeutic strategies to hinder compensatory signalling pathways. The interactions of resistant cells with sensitive cells are based on complex systemic networks. Figuring out the interactions between resistant cells and sensitive cells could provide new sights into the potential molecular mechanisms involved in resistance.

Accumulating researchers have found that ER stress confers the ability to cope with stress to cancer cells, thus resulting in drug resistance [23, 24]. In addition, it has been suggested that ER stress signal may be transmissible between cells in cancer, which can trigger many biological effects on nearby cancer cells, such as proliferation, invasion, drug resistance, etc [6, 24, 25]. Our study demonstrated that the occurrence of trastuzumab resistance was closely associated with ER stress in HER2-positive GC cells, and this resistance capacity also spread to recipient sensitive GC cells. However, we also found that the dissemination of trastuzumab resistance was not caused by the transmission of the UPR. Our findings are in line with those in previous research in which the genetic induction of a full UPR did not result in UPR transmission in co-culture [26].

Emerging evidence has indicated that exosomes from tumour cells could function as an essential regulator in tumour communication via transporting miRNAs to the recipient cells. Moreover, exosomal miRNAs regulated by ER stress are involved in many physiological and pathophysiological processes [26–28]. Nevertheless, where ER stressed GC cells could affect the trastuzumab sensitivity of unstressed GC cells via delivering exosomes and the resistance transfer is regulated by certain exosomal miRNAs remains unclear. Our research presented that ER stress significantly increased the release of exosomes from HER2-positive GC cells and that these exosomes effectively transmitted trastuzumab resistance to unstressed recipient cells, indicating that ER stress could influence the exosome secretion and mediated the transmission of trastuzumab resistance.

Accumulating studies have reported that the horizontal transporting of exosomal miRNAs led to drug resistance [18–20]. Although miR-301a-3p was overexpressed in various cancers and promoted tumour development and drug resistance through epigenetic or genetic mechanisms [29–32], the effects of exosomal miR-301a-3p and the underlying mechanisms by which miR-301a-3p regulates trastuzumab resistance still remain to be illustrated. Our study demonstrated that the miR-301a-3p level was elevated in ER stressed GC cells compared with unstressed GC cells and was functionally required for the trastuzumab resistance phenotype. We also discovered that miR-301a-3p was secreted from ER stressed GC cells via exosomes, which transformed trastuzumab sensitive cells into resistant cells and thereby disseminated trastuzumab resistance.

LRIG1 is highly related to tumour development in multiple cancers [33–36]. Besides, LRIG1 has an obvious negative feedback relation with RTKs [37, 38]. Previous research has demonstrated feed-forward regulating mechanisms in tumour cells in which abnormal HER2 signal reduced LRIG1 protein expression, which led to upregulated HER2 [39]. However, the level of HER2 amplification does not affect the trastuzumab sensitivity [40, 41]. FGFR1 can interact with fibroblast growth factors to activate downstream signalling pathways and has an essential part in the pathogenesis of GC [42, 43]. It has been reported that the activation of IGF-1R might indirectly influence the sensitivity of pan-HER2 inhibitors [21]. We discovered that LRIG1 expression in HER2-positive GC cells in response to trastuzumab was increased. However, its expression was still reduced in response to trastuzumab in ER stressed GC cells. We indicated that miR-301a-3p induced by ER stress promoted trastuzumab resistance via the direct binding relationship with the 3′-UTR of LRIG1, resulting in the downregulation of LRIG1 and subsequently facilitating the expression of IGF-1R and FGFR1. Furthermore, We also found that intercellular transfer of miR-301a-3p by exosomes from ER stressed GC cells disseminated the resistance phenotype to the unstressed recipient GC cells.

Our study revealed that miR-301a-3p induced by ER stress-mediated trastuzumab resistance via directly targeting LRIG1 to facilitate IGF-1R and FGFR1 expression. Moreover, intercellular transfer of miR-301a-3p by exosomes disseminated trastuzumab resistance. In conclusion, our results demonstrated that exosomal miR-301a-3p could be used as a non-invasive biomarker to indicate trastuzumab response, which was maybe a novel treating target to reduce trastuzumab resistance and improve the curative effect of trastuzumab in HER2-positive GC patients.

## Materials and methods

### Cell culture and treatments

The human-derived HER2-positive GC cell lines NCI-N87 and MKN45 were obtained from the American Type Culture Collection (ATCC). All cell lines in the following were recently authenticated before purchasing, and mycoplasma test was performed before all experiments. All cells were cultured in DMEM (Gibco, NY, USA) added with penicillin-streptomycin (1%) and FBS (10%) in a 37 °C incubator (5% CO_2_). Compounds were used to treat the cells for the specified time periods: 1 µM TG (Sigma, MO, USA) or -Glu/FBS.

### Cell transfection

The miR-301a-3p inhibitor, mimic, control vector and negative control (NC), LRIG1 siRNA and negative control siRNA (scramble) were synthesized by GenePharma (Shanghai, China). After the cell confluence reached 50–60%, the cells were transfected above mentioned recombinant plasmids by applying Lipofectamine™ 3000 reagent (Invitrogen, CA, USA). Cells were used for further experiments 72 h after transfection.

### Stable transfection with lentiviral vectors

After cells were cultured in the plate for 24 h (50,000 cells/ml), transfection was conducted by using 10 μl lentiviral vectors containing miR-301a-3p mimics, inhibitors, control vector or NC. Polybrene (4 μg/ml, Genechem, Shanghai, China) was used to enhance the transfection efficacy, and culture medium was changed 12 h after transfection. Puromycin (5 μg/ml Sigma-Aldrich) was used for selection after 4 days. Culture medium was replaced every 2 days and the selection was conducted for three times. qRT-PCR was used to confirm the efficiency of transfection.

### ER stress CM generation

Cells were treated with TG (1 µM) or an equal volume of vehicle (DMSO). Fresh, standard growth medium and Dulbecco′s PBS (Corning, VA, USA) were used to wash the cells after treatment for 6 h followed by incubation for 24 h. ERS CM was collected, followed by centrifugation for 10 min at 2000 rpm, and passed through a 0.22 µm filter (Millipore, MA, USA).

### Cell viability assay

The cells were cultured in a 96-well plate and treated by trastuzumab of different concentrations for 72 h. Then, CCK8 reagent was used to treat the cells for 2 h. The optical density was detected by using the spectrophotometer (Thermo Electron Corporation, MA, USA) at a wavelength of 450 nm. The percentage of viable cells was then calculated.

### Apoptosis assay

Cell apoptosis was assessed with Annexin V-FITC/PI staining kit (BD, USA). Cells were incubated with 3 μM OXA for 48 h post-transfection and harvested and stained with Annexin V–FITC for 15 min and PI for 5 min. The percentage of apoptotic cells was measured using LSR II flow cytometry (BD, USA).

### Western blot

Protein in exosomes or cells was obtained by using RIPA buffer (Sigma-Aldrich). SDS-polyacrylamide gel (10%) was used for electrophoresis. The protein was then transferred onto PVDF membranes (Millipore). The membranes were incubated overnight at 4 °C with primary antibodies, followed by incubation with HRP-linked secondary antibodies. A chemiluminescence substrate (Pierce Chemical, IL, USA) was used to visualize the protein band. All antibodies used are listed in Supplementary Table 1.

### RNA extraction and quantitative real-time PCR

Total RNAs in cells and exosomes were collected by applying the miRNeasy Micro Kit (Qiagen, CA, USA). cDNA was then synthesized by using SuperScript III Reverse Transcriptase (Invitrogen). qRT-PCR was conducted by utilizing Power SYBR Green (Takara, Dalian, China) and CFX96 Real-Time PCR Detection System (Bio-Rad, CA, USA). The primers of the microRNA used in this experiment were purchased from Ribobio (Guangzhou, China). The primers of mRNA are presented in Supplementary Table 2.

### Microarray analysis

miRNAs were collected from ~1 × 10^7^ cells by applying a miRNeasy Micro Kit following protocols. The miRNAs were labelled and hybridized in the Affymetrix GeneChip miRNA 4.0 Array (Affymetrix, Thermo Fisher Scientific), which included miRNAs in the miRBase v20 database (http://www.mirbase.org/). Affymetrix GeneChip Scanner 3000 was used to scan the microarray and miRNA QC Tool software (Affymetrix) was for analysis.

### Dual-luciferase reporter assay

Plasmid pGL3-LRIG1-3′-UTR or pGL3-LRIG1-3′-UTR-Mutation was synthesized (Supplementary Fig. S3a–b). ER stressed GC cells were transfected by 50 nM of miR-301a-3p or NC, 10 ng pGL3-LRIG1-3′-UTR or pGL3-LRIG1-3′-UTR-Mutation, and 1 ng pRL-TK by utilizing Lipofectamine™ 3000 reagent. After the transfection for 48 h, luciferase activity was examined with Dual-Luciferase Reporter Assay System.

### Phospho-receptor tyrosine kinase array

The proteome profiler human phospho-kinase array (R&D systems, ARY003B, MN, USA) was used to determine the phosphorylated protein profile. Whole-cell extracts were collected and incubated in the arrays of human phospho-kinase antibodies. Phosphorylation status was measured by incubation with anti-phosphotyrosine horseradish peroxidase following protocols.

### Exosomes purification and identification

After incubation in DMEM/F12 medium supplemented with 10% exosome-free FBS for 48 h, the medium was obtained. Exosome Precipitation Solution (System Biosciences, CA, USA) was applied to isolate the exosomes. The blood was collected in ethylenediaminetetraacetic acid (EDTA) containing collection tubes, and exoEasy Maxi Kit (Qiagen, Hilden, Germany) was used to isolate serum exosomes. The image of the exosomes was taken at 100 keV by using transmission electron microscopy (JEM-1-11 microscope, Tokyo, Japan). The Nanosight NS500 instrument (Malvern Instruments Ltd, Malvern, UK) and NTA 3.0 analytical software (Malvern Instruments Ltd) were used for quantification.

### In vivo xenograft and treatment experiments

All animal experiments followed the Guide for the Care and Use of Laboratory Animals (NIH publication no. 80-23, revised in 1996) and were in compliance with the institutional ethical guidelines. Athymic BALB/C nude mice (male, aged 6-weeks, SIPPR-BK Experimental Animal Co., China) were kept in standard pathogen-free environment. Animals were separated randomly. The investigators were blinded to the group allocation during the experiment and when assessing the outcome. A total of 5 × 10^6^ miR-301a-3p knockdown cells or control cells were subcutaneously injected into the mice. When the volume of the xenografts reached ~50 mm^3^, the mice were randomized for intratumoral injections of TG (0.25 µg/g body mass) or DMSO (1 ml) twice a week for 6 weeks. Trastuzumab (20 mg/kg) or an equal volume of control IgG was administered via tail vein injection twice a week for 6 weeks. The width (W) and length (L) of the tumours were measured with calipers. The volumes of the tumours were calculated by applying the formula: volumes = (L × W^2^)×0.5. This work was approved by the animal care and ethics committee of Southern Medical University.

### Exosome experiments in vivo

When the volume of the xenografts reached ~50 mm^3^, the mice were intratumorally injected with 5 µg exosomes twice per week for six weeks. Meanwhile, trastuzumab or IgG was given via the tail vein.

### IHC and ISH analysis

Paraffin-embedded sections deparaffinized, rehydrated, and placed in Tris buffer. Serum block was applied for 30 min at room temperature before incubation of the primary and secondary antibodies. HRP substrate was used for visualization, and sections were then counterstained with hematoxylin (Sigma Chemical Co, USA). The miR-301a-3p probes were double digoxigenin (DIG)-labelled mercury locked nucleic acid probes [miRCURY LNATM detection probes (Exiqon, Vedbaek, Denmark)]. ISH was performed according to the manufacturer’s protocol (Exiqon, Vedbaek, Denmark).

### Statistical analysis

Statistical analysis was performed by Student’s *t*-test (two-tailed) or one-way ANOVA. *P* < 0.05 was regarded as statistically significant. GraphPad Prism (provided by GraphPad Software, Inc., San Diego, USA) and SPSS 17.0 (purchased from SPSS Inc., USA) were used for all the statistical analyses. Average value ± SD (standard deviation) was used to express the data.

## Supplementary information

Supplemental tables

Supplementary figure 1

Supplementary figure 2

Supplementary figure 3

Supplementary figure 4

Supplementary figure 5

Supplementary figure legends

## Data Availability

The datasets used and/or analysed during the current study are available from the corresponding author on reasonable reques.
