# Supplementary material for: miR-301a-3p induced by endoplasmic reticulum stress mediates the occurrence and transmission of trastuzumab resistance in HER2-positive gastric cancer
Source: Cell Death Dis. 2021 Jul 13;12(7):696. doi: 10.1038/s41419-021-03991-3 (PMC8277821; doi:10.1038/s41419-021-03991-3)
Supplement: Supplementary file 1 — Supplemental tables [file 41419_2021_3991_MOESM1_ESM.docx]

**Supplementary Table 1. Primary antibodies used in this study**

| Antigens | Manufacturer | Application |
| --- | --- | --- |
| GRP78 | Cell Signaling Technology | 1:1000 for WB |
| P-AKT | Cell Signaling Technology | 1:1000 for WB |
| AKT | Cell Signaling Technology | 1:1000 for WB |
| p-ERK | Cell Signaling Technology | 1:1000 for WB |
| ERK | Cell Signaling Technology | 1:1000 for WB |
| LRIG1 | Abcam | 1:1000 for WB |
| IGF-1R | Invitrogen | 1:1000 for WB |
| FGFR1 | Abcam | 1:1000 for WB |
| HER2 | Cell Signaling Technology | 1:1000 for WB |
| p-Ago2 | ECM Bioscience | 1:1000 for WB |
| CD63 | Proteintech | 1:1000 for WB |
| CD81 | Proteintech | 1:1000 for WB |
| Calnexin | Proteintech | 1:1000 for WB |
| GAPDH | Cell Signaling Technology | 1:5000 for WB |
| β-actin | Cell Signaling Technology | 1:5000 for WB |

**Supplementary Table 2. Primer sequences for qRT-PCR in this study**

| Genes | Primer sequences |
| --- | --- |
| miR-301a-3p | F: 5ʹ-ACACTCCAGCTGGGCAGTGCAATAGTATTGTC-3ʹ  R: 5ʹ-CTCAACTGGTGTCGTGGA-3ʹ |
| miR-509-3p | F: 5'-TGATTGGTACGTCTGTGGGTAG-3'  R: 5ʹ-CTCAACTGGTGTCGTGGA-3ʹ |
| miR-34c-5p | F: 5'-AGGCAGTGTAGTTAGCTGATTGC-3'  R: 5ʹ-CTCAACTGGTGTCGTGGA-3ʹ |
| miR-17-5p | F: 5'-CGGCGGCAA AGTGCTTACAG-3'  R: 5ʹ-CTCAACTGGTGTCGTGGA-3ʹ |
| miR-23a-3p | F: 5'- AUCACAUUGCCAGGGAUUUC-3'  R: 5ʹ-CTCAACTGGTGTCGTGGA-3ʹ |
| miR-8084 | F: 5'-ACGCGCGAATACTAAGTAAAAAA-3'  R: 5ʹ-CTCAACTGGTGTCGTGGA-3ʹ |
| miR-27a-3p | F: 5'-CGCGTTCACAGTGGCTAAGT-3'  R: 5ʹ-CTCAACTGGTGTCGTGGA-3ʹ |
| miR-374b | F: 5'-TCAGCGGATATAATACAACCTGC-3'  R: 5ʹ-CTCAACTGGTGTCGTGGA-3ʹ |
| miR-15b-5p | F: 5'-TGGAATTGACTTGGACCATAATAGA-3'  R: 5ʹ-CTCAACTGGTGTCGTGGA-3ʹ |
| miR-4428 | F: 5'-GCAAGGAGACGGGAACA-3'  R: 5ʹ-CTCAACTGGTGTCGTGGA-3ʹ |
| miR-35-3p | F: 5'-CATTGCACTTGTCTCGGTCTGA-3'  R: 5ʹ-CTCAACTGGTGTCGTGGA-3ʹ |
| U6 | F: 5ʹ-CTCGCTTCGGCAGCACA-3ʹ  R: 5ʹ-AACGCTTCACGAATTTGCGT-3ʹ |
| LRIG1 | F: 5ʹ-TTGAGGACTTGACGAATCTGC-3ʹ  R: 5ʹ-CTTGTTGTGCTGCAAAAAGAGAG-3ʹ |
| IGF-1R | F: 5ʹ-GGCACAATTACT GCTCCA AAGAC -3ʹ  R: 5ʹ- CAAGGC CCT TTCTCCCCA C -3ʹ |
| FGFR1 | F: 5ʹ- GGCTACAAGGTCCGTTATG -3ʹ  R: 5ʹ- CAATCTTACTCCCATTCACC -3ʹ |
| HER2 | F: 5ʹ- CATGTCATCGTCCTCCAGCAG -3ʹ  R: 5ʹ- TTGACTCTGAATGTCGGCCAA -3ʹ |
| GAPDH | F: 5ʹ-AGGTCGGTGTGAACGGATTTG-3ʹ  R: 5ʹ-TGTAGACCATGTAGTTGAGGTCA-3ʹ |
