# Supplementary figures and images for: miR-301a-3p induced by endoplasmic reticulum stress mediates the occurrence and transmission of trastuzumab resistance in HER2-positive gastric cancer
Source: Cell Death Dis. 2021 Jul 13;12(7):696. doi: 10.1038/s41419-021-03991-3 (PMC8277821; doi:10.1038/s41419-021-03991-3)

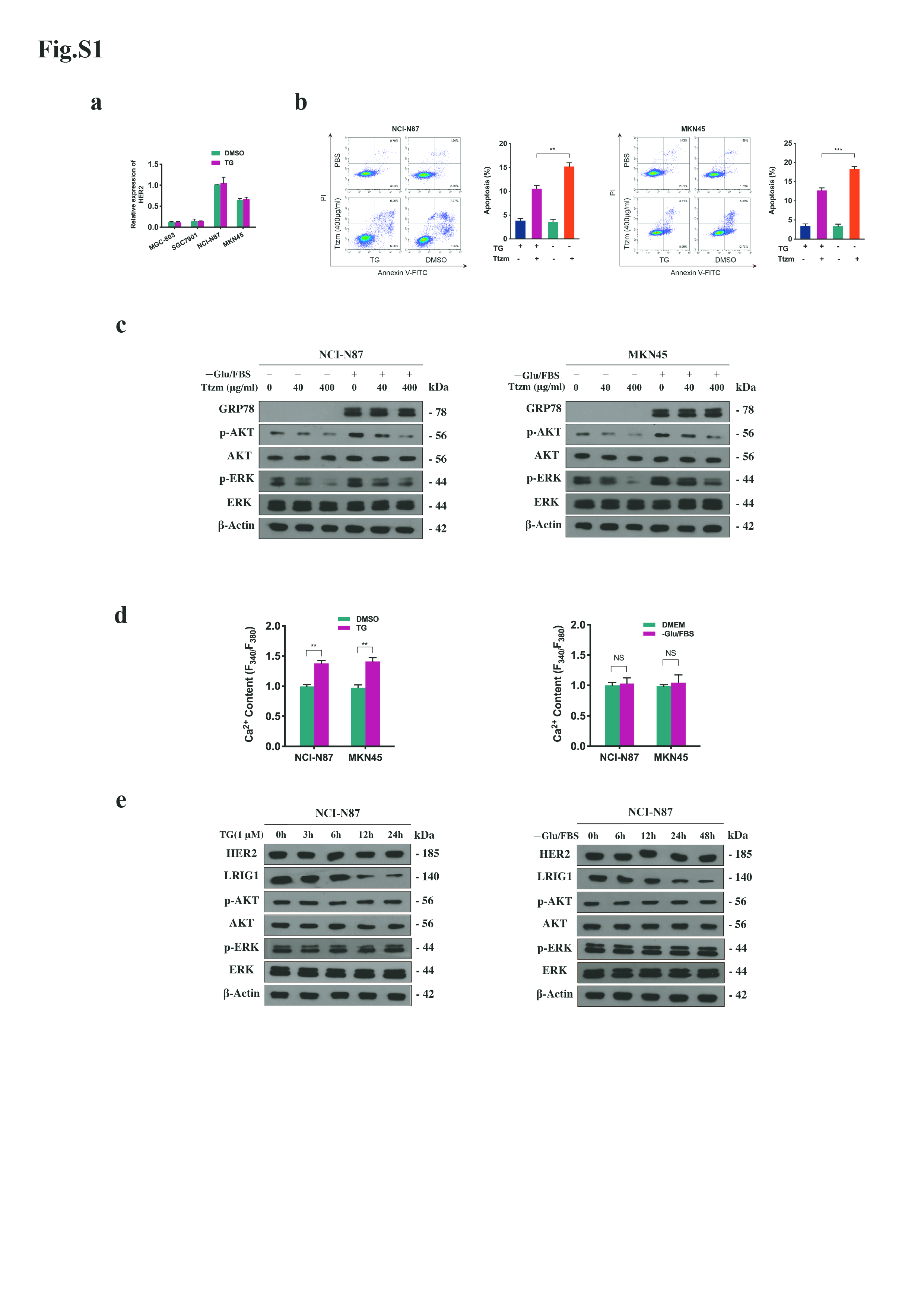

Supplement: Supplementary file 2 — Supplementary figure 1 [file 41419_2021_3991_MOESM2_ESM.tif]

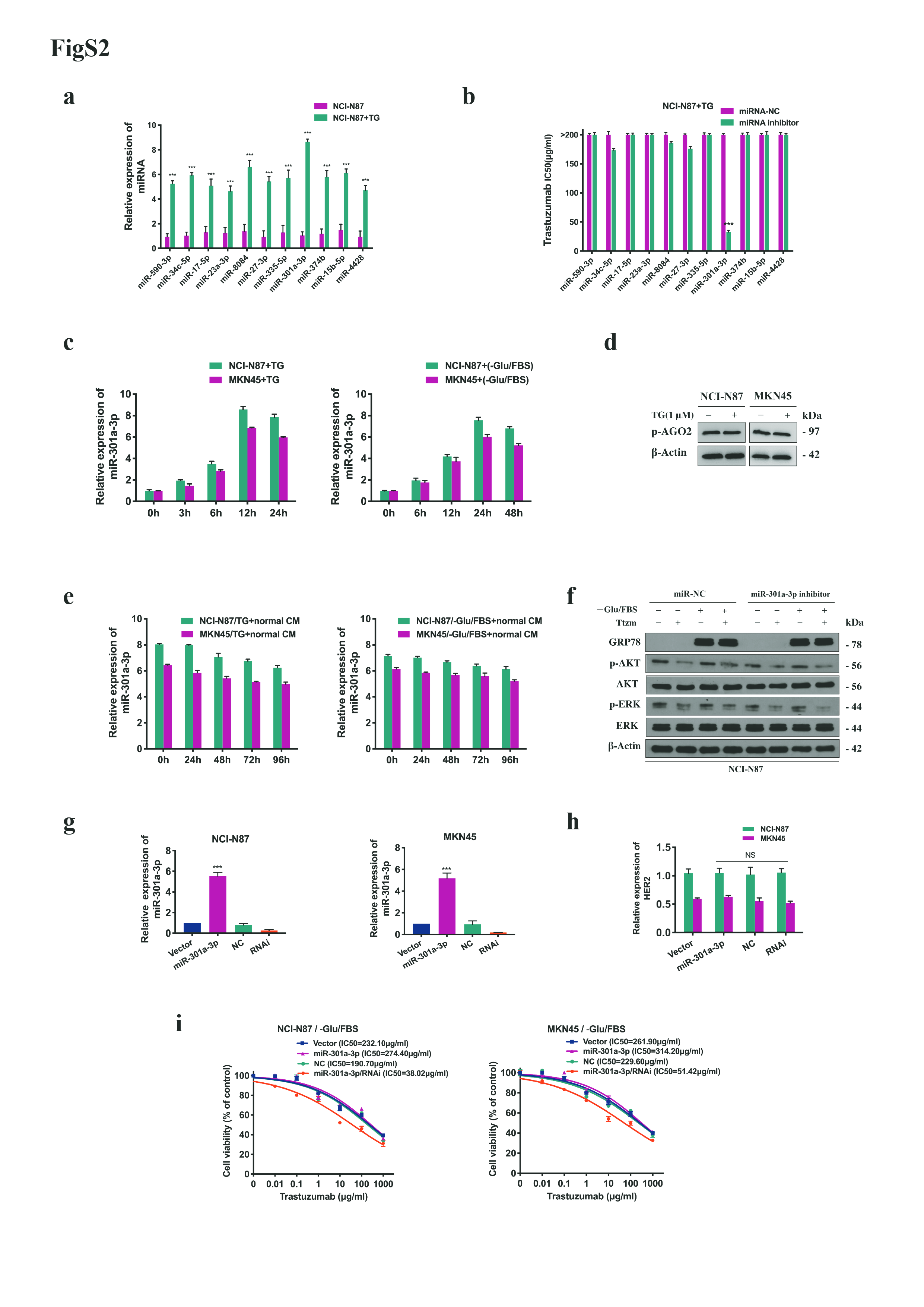

Supplement: Supplementary file 3 — Supplementary figure 2 [file 41419_2021_3991_MOESM3_ESM.tif]

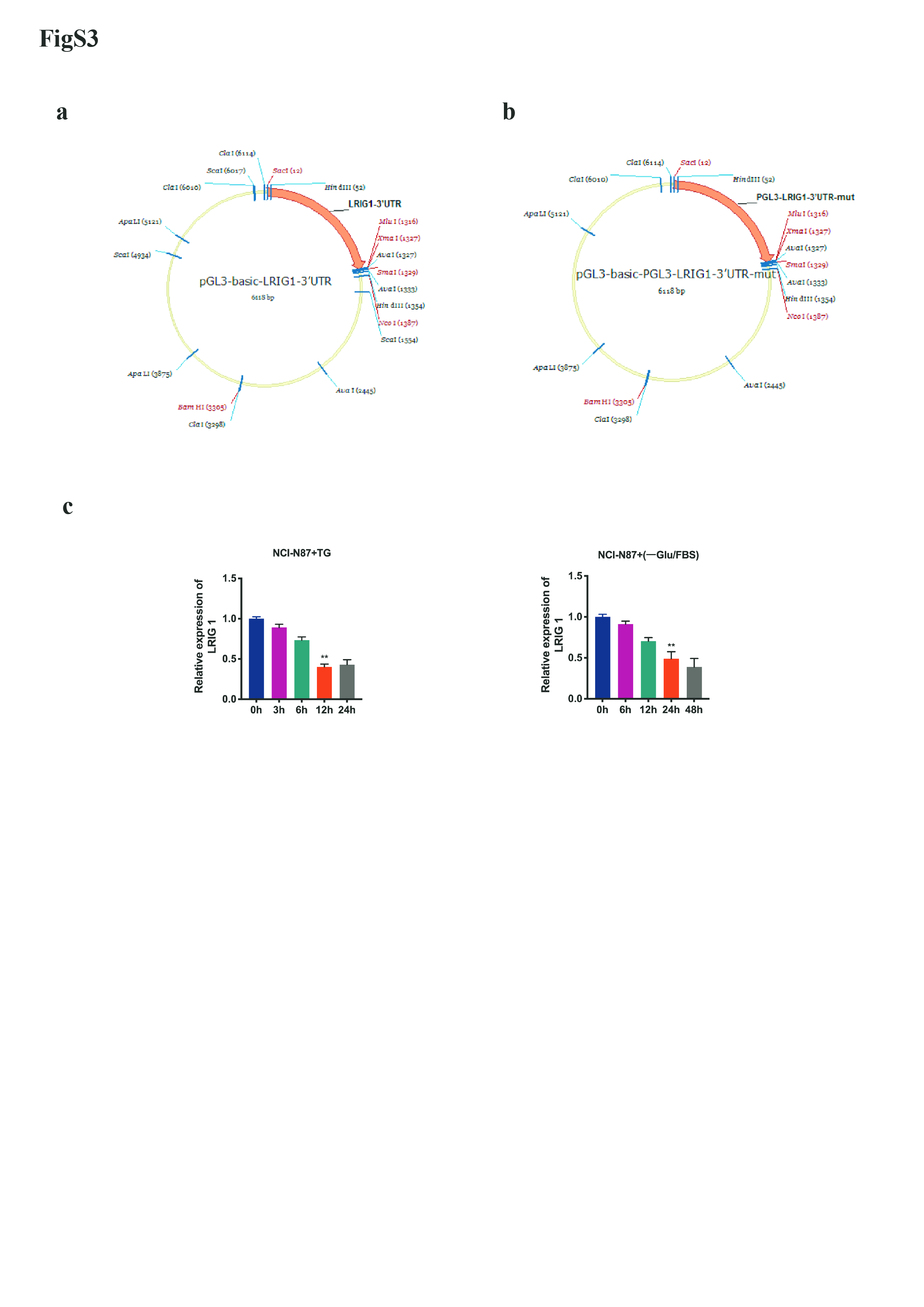

Supplement: Supplementary file 4 — Supplementary figure 3 [file 41419_2021_3991_MOESM4_ESM.tif]

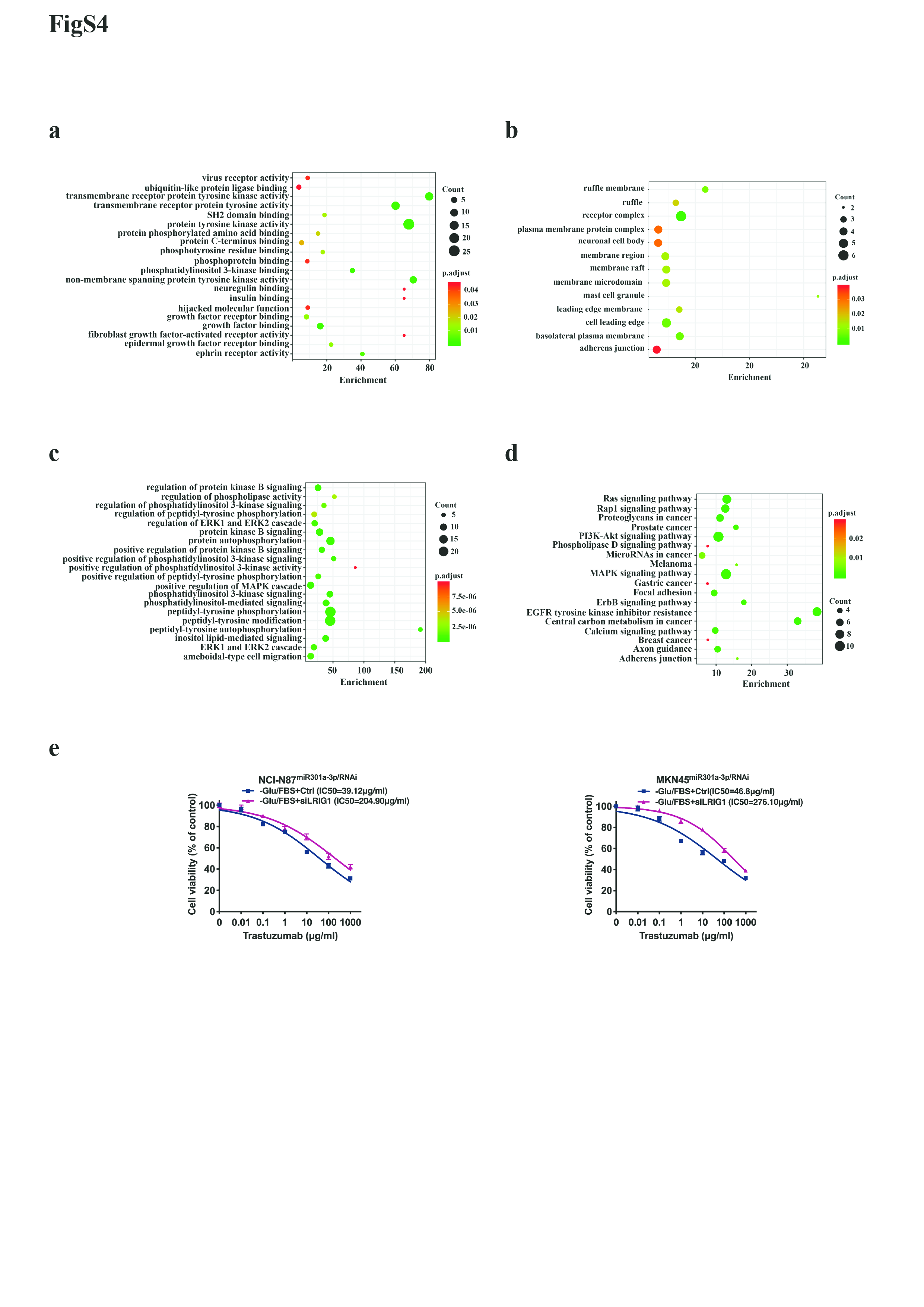

Supplement: Supplementary file 5 — Supplementary figure 4 [file 41419_2021_3991_MOESM5_ESM.tif]

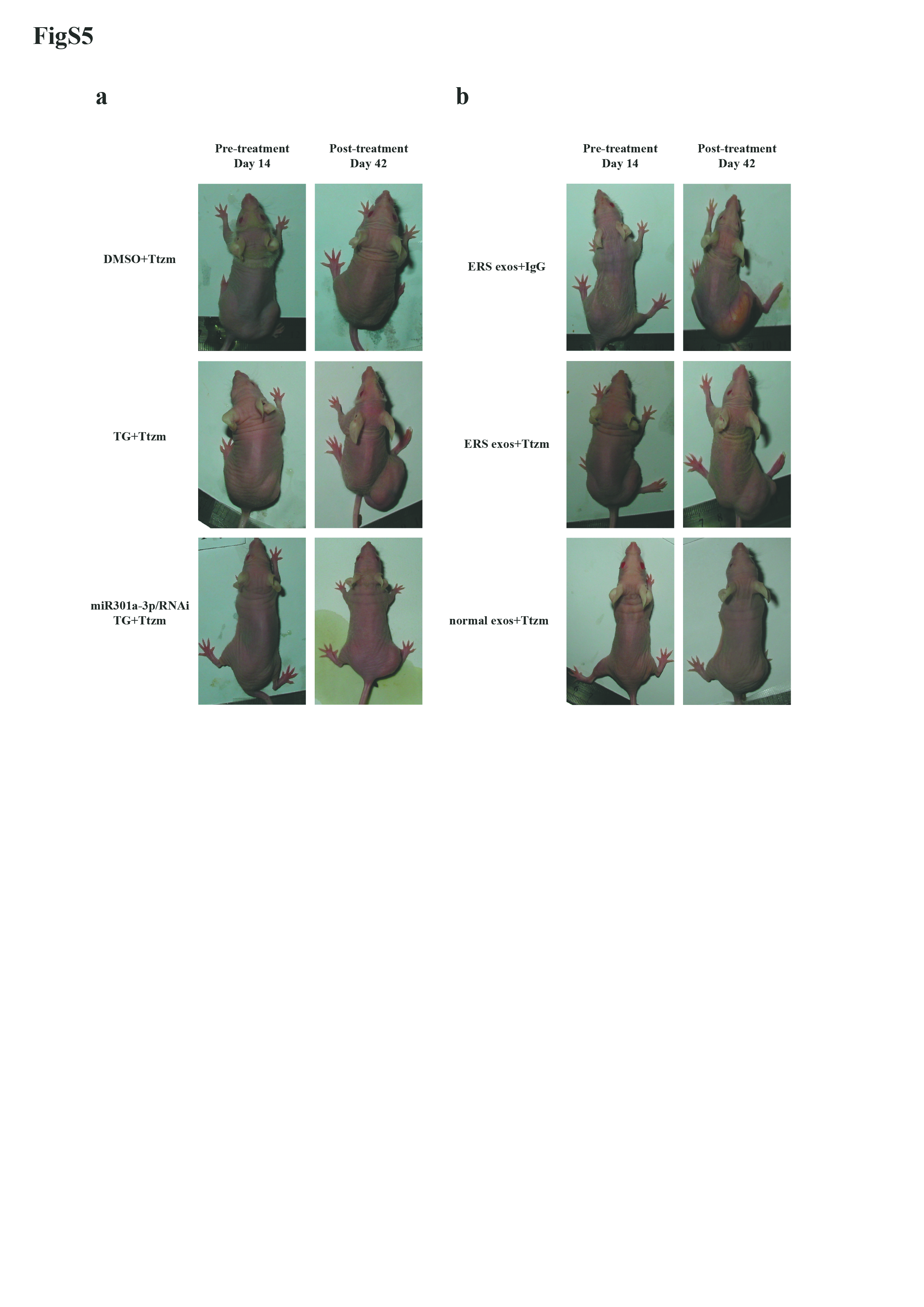

Supplement: Supplementary file 6 — Supplementary figure 5 [file 41419_2021_3991_MOESM6_ESM.tif]
