# Supplementary material for: miR-301a-3p induced by endoplasmic reticulum stress mediates the occurrence and transmission of trastuzumab resistance in HER2-positive gastric cancer
Source: Cell Death Dis. 2021 Jul 13;12(7):696. doi: 10.1038/s41419-021-03991-3 (PMC8277821; doi:10.1038/s41419-021-03991-3)
Supplement: Supplementary file 7 — Supplementary figure legends [file 41419_2021_3991_MOESM7_ESM.docx]

**Supplementary figure legends**

**Fig.S1. ER stress mediated trastuzumab resistance.**

(a) qRT-PCR analysis of HER2 level in HER2-positive (NCI-N87, MKN45) and HER2-negative (MGC-803, SGC7901) GC cells after treated with 1μM TG for 12h (n=3).

(b) Flow cytometry was used to measure the apoptosis of NCI-N87 and MKN45 cells after treated with 1μM TG for 12h followed by 400µg/ml trastuzumab treatment for 72h (n=3).

(c) Western blot analysis for indicated proteins in NCI-N87 and MKN45 cells incubated with -Glu/FBS for 24h followed by 40 or 400µg/ml trastuzumab treatment for 72h.

(d) Fura-2/AM was used to measure the intracellular Ca2+ contents in NCI-N87 and MKN45 cells after treated with 1μM TG for 12h or -Glu/FBS for 24h (n=3).

(e) Western blot analysis for indicated proteins in NCI-N87 cells after treated with 1μM TG for 0, 3, 6, 12 and 24h or -Glu/FBS for 0, 6, 12, 24 and 48h, respectively.

Statistical analysis was performed by Student′s t-test, error bars indicate SD (***p* < 0.01, ****p*< 0.001).

**Fig.S2. MiR-301a-3p mediated trastuzumab resistance.**

(a) qRT-PCR demonstrated that miRNAs were differentially expressed in NCI-N87 cells treated with or without 1μM TG for 12h (n = 3).

(b) The IC50 of trastuzumab was determined by CCK8 assay in NCI-N87 cells transfected with indicated miRNA inhibitors or negative control for 72h followed by 1μM TG treatment for 12h (n = 3).

(c) qRT-PCR analysis of miR-301a-3p expression in NCI-N87 and MKN45 cells after treated with 1μM TG for 0, 3, 6, 12 and 24h or -Glu/FBS for 0, 6, 12, 24 and 48h, respectively.

(d) Western blot analysis for p-AGO2 expression in NCI-N87 and MKN45 cells after treated with 1μM TG for 12h.

(e) qRT-PCR analysis of miR-301a-3p expression in NCI-N87 and MKN45 cells after treated with 1µM TG for 12h or -Glu/FBS for 24h following culture in normal CM (n = 3).

(f) Western blot analysis for indicated proteins in NCI-N87 cells after transfection with miR-301a-3p inhibitors or control. The cells were treated with -Glu/FBS for 12h followed by trastuzumab treatment for 72h.

(g) qRT-PCR analysis of miR-301a-3p expression in miR-301a-3p stably transfected and control NCI-N87 and MKN45 cells (n = 3).

(h) qRT-PCR analysis of HER2 expression in miR-301a-3p stably transfected and control NCI-N87 and MKN45 cells (n = 3).

(i) CCK8 assay of miR-301a-3p stably transfected and control NCI-N87 and MKN45 cells. The cells were incubated with -Glu/FBS for 24h followed by trastuzumab treatment for 72h (n = 3).

Statistical analysis was performed by Student′s t-test, error bars indicate SD (****p*< 0.001).

**Fig.S3. The structure of recombinant plasmids.**

The structure of recombinant plasmids including pGL3-LRIG1-3′-UTR (a) and pGL3-LRIG1-3′-UTR-Mut (b).

(c) qRT-PCR analysis of LRIG1 expression in NCI-N87 cells after treated with 1μM TG for 0, 3, 6, 12 and 24h or -Glu/FBS for 0, 6, 12, 24 and 48h, respectively (n=3).

Statistical analysis was performed by Student′s t-test, error bars indicate SD (***p* < 0.01).

**Fig.S4. GO and KEGG analysis of the DEPs.**

Protein function annotation GO and KEGG functional enrichment analysis of the identified DEPs. GO analysis included 3 subtypes: (a) molecular function (MF), (b) biological process (BP) and (c) cellular component (CC). (d) KEGG is systematic analysis for gene functions. y-axis represents GO terms; x-axis represents enrichment. The color of each bubble represents the *P* value, and the bubble size represents the gene count.

(e) CCK8 assay of miR-301a-3p knockdown NCI-N87 and MKN45 cells transfected with siLRIG1 or control and incubated with -Glu/FBS for 24h followed by trastuzumab treatment for 72h (n = 3).

**Fig.S5. The representative images of nude mice.**

(a) Nude mice were subcutaneously injected with miR-301a-3p stable knockdown or control NCI-N87 cells. When the volume of xenografts grew to about 50 mm^3^ after 14 days, the mice received intratumoral injections of TG (0.25µg/g body mass) or DMSO and were treated with trastuzumab (20mg/kg) or an equal volume of control IgG via tail vein injection twice a week for 6 weeks (n = 5 per group). The representative images of nude mice in the indicated group are shown.

(b) Nude mice were subcutaneously injected with NCI-N87 cells. When the volume of xenografts grew to about 50 mm^3^ after 14 days, the mice were intratumorally injected with 5µg ER stressed exosomes or normal exosomes, and treated with trastuzumab (20mg/kg) or an equal volume of control IgG via tail vein injection twice a week for 6 weeks (n = 5 in each group). Representative images of nude mice were shown.
